# Retreatment of Gingival Recessions: Rationale and Definition Case Series Follow-Up

**DOI:** 10.1155/ijod/6685763

**Published:** 2025-09-08

**Authors:** Alon Sebaoun, Perry Raz, Liat Chaushu, Michael Saminsky

**Affiliations:** Department of Periodontology and Oral Implantology, Goldschleger School of Dental Medicine, Gray Faculty of Medical and Health Sciences, Tel Aviv University, Tel Aviv, Israel

**Keywords:** case series, gingival recession, retreatment, root coverage

## Abstract

**Aims:** One of the main objectives of root coverage procedures is complete coverage of the denuded root surface. If this goal is not obtained, retreatment may be considered.

**Materials and Methods:** This retrospective case series study evaluated the need for surgical retreatment among 105 gingival recessions, in 56 patients. Pre- and postretreatment recession depth (RD), keratinized tissue (KT) width, and attachment loss (AL) were extracted from follow-up registries.

**Results:** A total of 17 recessions (11 Miller Class I-II/RT1 and 6 Miller Class III/RT2) in 13 patients (nine and four, respectively) were retreated surgically. The mean follow-up was 2.2 years (range 1–9). RD and the clinical attachment level gain (CAL gain) improved significantly after retreatment (*p*=0.0017). The linear regression model revealed a significantly higher KT gain (KTG) and reduction in residual RD (RRD), in patients with initial Miller III/RT2 recessions, when followed up for more than 1 year (*p* < 0.0001).

**Conclusions:** The present results indicate that a root recession retreatment procedure is a viable option with high predictability and long-term stability.

## 1. Introduction

Gingival recession is the apical displacement of the gingival margin from the cementoenamel junction (CEJ) with oral exposure of the root surface [1]. According to previous reports, untreated gingival recessions are very likely to progress over time [2]. Different techniques to obtain predictable and esthetic root coverage in a wide variety of clinical situations have been described [3]. Treatment of soft tissue marginal recessions aims for complete root coverage (CRC), with an esthetic and natural appearance of the new tissue [4]. According to a systematic review, [5] the average percentage of root coverage for Miller Class I [6] and II/RT1 [7] recessions is 80.9%, with a range of 50%–97.3%. Notably, another report described mean and range values of 70% (34%–87%) [8]. Although Miller Class I and II/RT1, recessions are most usually studied, there are also reports that a range of 54.8%–85% of root coverage can be achieved for Miller Class. III/RT2 recessions [5]. Whereas CRC can be expected for Class I and II gingival recessions, only partial results may be possible for Class III/RT2 [3, 8]. Various factors may contribute to the failure of root coverage procedures. These can be broadly categorized into patient-related factors (e.g., smoking and inadequate oral hygiene practices), site-related factors (such as soft tissue thickness and amount of keratinized tissue (KT) (gingival phenotype), and surgical-related factors (including improper surgical technique, excessive flap tension, or perforation, and instability of the flap or graft) [9–12]. A successful result should remain stable over time. However, an apical shift of the gingival margin is observed in about 40% of treated patients, who experience progressive gingival recession with time [13]. Although surgical retreatment may be a viable solution for such cases, scientific literature is relatively scarce on this topic [14]. Moreover, the term “retreatment” in root coverage procedures has not been clearly defined. This retrospective study presents a clinical case series of retreatments of gingival recessions and an examination of their stability over time. Additionally, a definition for recession coverage retreatment is proposed.

## 2. Materials and Methods

Between 2005 and 2015, a root recession coverage procedure was performed in 56 patients (105 teeth). All patients were treated and followed in a private practice setting by the same periodontist (Alon Sebaoun). The total cohort comprised 11 males, aged 18–41 (mean 30.7) and 45 females, aged 17–51 (mean 31.7). Thirty-three patients had a single recession, 21 had multiple treated recessions, and two patients were treated for multiple and single recessions. As measured from the gingival margin to the CEJ, recession size ranged between 2 and 10 mm. Eighty-two recession defects were Miller Cl I, II/RT1, and 23 were classified as Miller Cl-III/RT2. Different well-documented techniques were applied [14–20].

A second surgery was performed in cases where the patient was dissatisfied with the esthetic (e.g color mismatching with surrounding tissues) or functional results (e.g., thermal root sensitivity, discomfort while toothbrushing) over a timeframe of 8 weeks to 2 years post-op. The decision to proceed with a second surgery was also based on clinical criteria, including insufficient KT width or thickness (gingival phenotype) achieved after the initial procedure, or when the clinical outcome was equal to or worse than the baseline condition. The following clinical parameters were recorded prior to the surgery and at the follow-up visits:

• Recession depth (RD) from the CEJ to the gingival margin at the central buccal site.
• KT width from the gingival margin to the mucogingival junction.
• Attachment loss (AL) which is a summation of probing depth and RD.

The obtained data were used to calculate the residual RD (RRD), KT gain (KTG), and clinical attachment level gain (CAL gain). All measurements were assessed by the same operator (AS) using a UNC-15 periodontal probe (Hu-Friedy). Measurements were recorded to the nearest millimeter. The surgical results were evaluated 3, 6, and 12 months after the procedure. Further follow-ups were conducted annually.

This study is a retrospective case series study and was performed in a private practice setting. The study protocol was reviewed and approved by the Tel-Aviv University Ethics Committee (Ref.: 0008444-3). This case series is reported in accordance with the PROCESS Guideline [21].

### 2.1. Statistical Analysis

As preliminary univariate analysis, Mann–Whitney test was used to test differences in RD, KT, and AL between Miller I/II (RT1) and Miller III (RT2) recessions at 1-year vs. later follow-up visits. No statistically significant or close to significant trends were detected for RD measured prior to the first surgery or AL (*p* >  0.1), and therefore a related-samples Wilcoxon signed rank test was applied. In contrast, statistically significant, and close to significant differences were detected for KT and RD measured prior to “retreatment,” and therefore a linear regression model was used to examine differences in KT before and after retreatment, with Miller groups (I/II vs. III) and follow-up groups (1-year vs. >1 year) as covariates. Effect sizes and their confidence intervals were computed for the tests. The confidence intervals were entirely above 0, suggesting a significant effect. *p*-Values were corrected for multiple comparisons using the Benjamini–Hochberg (BH) procedure. The observation that four patients each contributed two teeth to the study could make a mixed model more suitable for the analysis. However, instead we chose to randomly disregard one tooth for each of these patients. The level of significance used in the analyses was 5% (*α* = 0.05). Statistical analysis used SPSS Version 29.0.1.0 and R Version 4.4.0 statistical software.

## 3. Results

Eighteen patients were not satisfied with the results of their initial surgery, and retreatment was proposed. One patient refused a second surgery, but 17 patients (14 females and 3 males) underwent root coverage retreatment. All participants except one were otherwise healthy and nonsmokers. The mean age was 30.59 (21–50). Eleven recessions (13%) that were initially classified as Miller I, II/RT1 in nine patients, and six recessions (26%) classified as Miller III/RT2, in four patients, failed to reach 70% or 50% coverage, respectively. The type of second surgery chosen for correction was based on the thickness and quality of the tissue apical and lateral to the recession (Table 1) [23]. The mean follow-up after the second surgery was 2.2 (1–9) years. Clinical parameters assessed prior to surgical treatment and after “retreatment” are presented in Table 2. RRD was significantly lower after retreatment, while compared to RD measured prior to the first surgery (*p*=0.0017, Effect size = 25.24, CI: [2, 4.07], Wilcoxon Signed Rank Test, BH corrected). Similarly, there was a statistically significant AL gain while compared to attachment level measures prior to both first (*p*= 0.0017, Effect size = 25.24, CI: [2.04, 4.27], Wilcoxon Signed Rank Test, BH corrected), and “retreatment” surgeries (*p*= 0.0017, Effect size = 24.96, CI: [0.74, 4.53], Wilcoxon Signed Rank Test, BH corrected). A linear regression model of KTG (constant), considering Miller Class/RT, and follow-up period as covariates was statistically significant compared to both first (*p* <  0.0001, BH corrected, *B* = 2.33, CI: [1.68, 2.99]), and “retreatment” surgeries, respectively (*p* <  0.0001, BH corrected, *B* = 1.56, CI: [0.85, 2.26]). Likewise, a linear regression model for RD, also incorporating Miller Class/RT and follow-up period as a covariate, demonstrated a statistically significant difference between RD measured prior to retreatment and the RRD; (*p*  < 0.0001, BH corrected; *B* = −2.78, CI: [−3.59, −1.96]). In all cases >70% recession coverage was achieved. Three clinical cases of pre and postgingival recession retreatment are presented.

### 3.1. Clinical Retreatment Cases

#### 3.1.1. Case 1 Patient Presentation (Figure 1A–H)

A systemically healthy, nonsmoking, 26-year-old woman presented with single tooth recessions in different regions of the mouth (13, 23, 44, and 31). These were all Miller Class I and II/RT1 recessions. The patient was referred for a preorthodontic periodontal evaluation. After clinical and radiographic evaluation, the patient was advised to proceed with preorthodontic root coverage and phenotype modification procedures [24]. Supragingival plaque was removed, and oral hygiene instructions were given before surgery. The recessions in teeth 13, 23, and 44 were treated with the connective tissue and partial thickness pedicle graft technique [11], while the modified tunnel double papilla technique [16] was used for the lower incisors. As a frenulum pull was noticed, a frenectomy–vestibuloplasty was performed 10 weeks before the root coverage procedure to reduce tension [25]. At the time of suture removal (10 days), immediate total failure was observed. The patient was informed, and a decision for retreatment was postponed for at least 2 months. After 6 months, the tissue appeared completely mature, and a retreatment was scheduled. The gingival environment lateral to the recession appeared thicker than before the first surgery, which allowed us to use the connective tissue and partial thickness pedicle graft technique, [17] combined with the tunnel technique at the adjacent teeth. Full coverage with good color blending and stability were visible at 2-month and 1-year post-op visits.

#### 3.1.2. Case 2 Patient Presentation (Figure 2A–H)

A 50-year-old otherwise healthy woman presented with multiple deep Miller III/RT2 recessions in the lower jaw and a complaint of pain in one of the recessions during tooth brushing. Clinical examination revealed clinical signs of inflammation at the level of the gingival margin of tooth 42 and deep recessions on teeth 42, and 43, with a lack of attached gingiva and thin periodontal phenotype tissue. Supragingival plaque was removed, and oral hygiene instructions for the use of a super soft toothbrush were provided before surgery. A root coverage procedure using the tunnel technique [16] was used, with a connective tissue graft harvested from the right tuberosity by a distal wedge procedure. The graft was split in two [26] to enable coverage of the two teeth (42 and 43) and was then placed and fixed in the previously prepared tunnel. The results at 2- and 5-months post-op showed less than 50% root coverage and tissue thickening. Despite the appearance of healthy gingival tissue, the patient agreed with the decision to perform a second surgery to attempt a better result. A similar surgical procedure used a connective tissue graft harvested from the left tuberosity by a distal wedge procedure. Results after 3 months, 1 year, and 6 years reveal almost complete and stable root coverage with healthy and thickened gingiva.

#### 3.1.3. Case 3 Patient Presentation (Figure 3A–H)

A systemically healthy, 27-year-old woman presented with two adjacent recessions in the anterior lower jaw. Clinical examination revealed a 7 mm and a 4 mm recession classified as Miller III/RT2, and very thin tissue in teeth 31 and 41, respectively. Supragingival plaque was removed, and oral hygiene instructions were provided before surgery. A frenulum pull was noticed, and frenectomy–vestibuloplasty was performed 8 weeks before the root coverage procedure [24]. A root coverage procedure [20] was performed with a connective tissue graft harvested from the lateral palate after de-epithelialization in situ [27]. After 3 months of healing, the tissue appeared thicker, but the root was less than 50% covered. The procedure for the second surgery was the modified tunnel double papilla technique [16] with connective tissue graft harvested from the lateral palate after de-epithelialization in situ [27]. The results after 2 months and 3 years reveal an almost complete and stable root coverage with enlargement and thickening of the attached gingiva.

## 4. Discussion

Any treatment should have the goal of fulfilling patient expectations. The goals after the root coverage procedure are both functional and esthetic, which translates to CRC and accurate color blending [28]. Root coverage results should remain stable for at least 5 years, which can be achieved through periodontal soft tissue phenotype modification. In a recent longitudinal study, the presence of at least 1.5 mm of KT and 1.46 mm gingival thickness could be correlated with long-term stability (10 years) of the gingival margin following the root coverage procedure [29]. CRC after treatment of Miller Class I and II/RT1 recession defects was reported in 46.6% (7.7%–91.6%) of cases [4, 7]. Another study reported CRC in 35% (15%–60%) of Miller Class I and II/RT1 recession defects [8]. In contrast, CRC was obtained in 38%–57% of Class III/RT2 recessions [30, 31]. These finding reveal that the goal of CRC is not always achieved. The question of whether and what the chances are that a second surgery can provide CRC requires investigation.

Although the desirable root coverage was not achieved after the first surgery in these cases, periodontal phenotype modification, as manifested by thickening of the tissues and enlargement of the KT width, was observed, and this may have assisted the success of the second surgery. The “retreatment” procedure achieved the desired outcome, and the results were stable up to 9 years of follow-up.

Interestingly, the logistic regression analysis revealed a significantly higher KTG in cases followed up for more than 1 year. This may correspond to previous findings of the “creeping attachment” phenomenon, [32] which was observed after long-term follow-up [33]. There were also statistically significant improvements in the clinical parameters of RRD and AL gain after the retreatment procedure. These findings strengthen the applicability of this clinical approach.

The criteria for retreatment of gingival recession defects have not been clearly defined. Inspired by the endodontic literature concerning root canal retreatment, [34] the following definition for retreatment of gingival recession may be suggested: A surgical procedure designed to correct previously treated recession defects that exhibit an unsatisfactory outcome due to surgical complications and/or patient-induced mechanical trauma (e.g., incorrect tooth brushing technique).

The present study was performed in a private practice setting. Long-term evaluation of root coverage procedures results requires extensive documentation followed by statistical analysis, both of which are usually performed in academic settings. This may explain the relative scarcity of data from private periodontal practices, and especially when considering retreatment procedures. However, when available, these data are particularly valuable in extending our understanding of the applicability of this clinical approach.

The clinical significance of our findings lies in their potential to assist clinicians in making more informed decisions when planning root coverage retreatments. Although each case demands a personalized approach based on anatomical variations—such as the characteristics of the surrounding soft and hard tissues and the patient's gingival phenotype—our results contribute to a better understanding of the factors involved in managing such scenarios. One important clinical consideration is determining which surgical technique to apply in a retreatment scenario: should the previously used technique be repeated despite its failure, or should an alternative approach be chosen? In our opinion, this decision should primarily be guided by the current condition of the tissues following the initial procedure, as well as the clinician's experience and proficiency with various evidence-based root coverage techniques. While tunneling procedures combined with connective tissue grafts, or pedicle and advanced flap techniques, may offer specific advantages depending on the clinical situation, we emphasize that the optimal choice should be tailored to the individual case. By integrating these considerations, clinicians can enhance predictability and outcomes in complex retreatment cases. Future studies analyzing a larger population of patients undergoing gingival recession retreatment procedures may offer valuable information for the clinicians.

## 5. Conclusions

Based on the investigated clinical case series of root recession retreatment, we can conclude that these procedures are a viable option, with predictable results. Factors such as surrounding tissue thickness and quality apical and lateral to the recession will determine the type of second surgical procedure to be used.

## Figures and Tables

**Figure 1:**
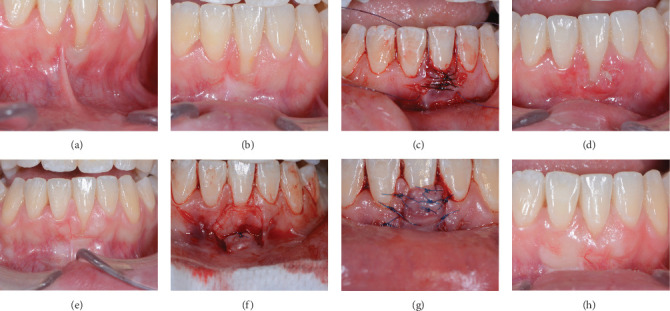
(A) Preoperative aspect of tooth 31 with Miller class II/RT1 recession. Note frenum pull. (B) Around 10 weeks after vestibuloplasty and before the root coverage procedure. Note the lack of tension from the frenulum and some reduction in the dimensions of the recession. (C) Modified tunnel double papilla procedure (MTDP) [16]. (D) Around 10 days after surgery. Immediate total failure may be observed. (E) Around 6 months after the first surgery and before retreatment. Note thicker gingival environment lateral to the recession. (F) Retreatment surgery—The connective tissue and partial thickness pedicle technique [15] combined with the tunnel technique. Note the connective tissue graft insertion within the tunnel created lateral to the recession. (G) The double pedicle covers the connective tissue graft and is stabilized coronal to the CEJ. (H) Around 12 months postoperative result. Note the complete root coverage and phenotype modification.

**Figure 2:**
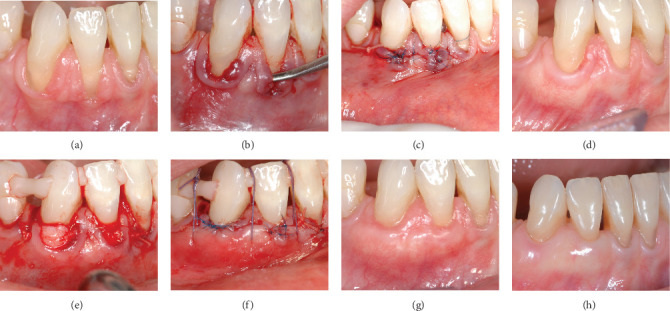
(A) Preoperative view of Miller class III/RT2 gingival recession. Note the lack of attached gingiva and injury of the gingival margin (42). (B) Tunnel preparation, around teeth 42, 43, for the modified tunnel double papilla technique (MDTP) [16]. (C) Closed tunnel over connective tissue graft harvested from the tuberosity. (D) Results at 5 months after the first surgery. Partial root coverage and thickening of the tissue can be seen. (E) Retreatment surgery—Connective tissue graft from the tuberosity [26] inside the prepared tunnel around teeth 42, 43. (F) End of the second surgery with vertical mattress sutures in place. (G) Results at 12 months post-op, showing almost complete root coverage and phenotype modification. (H) Results at 6 years showing long-term stability of the obtained results.

**Figure 3:**
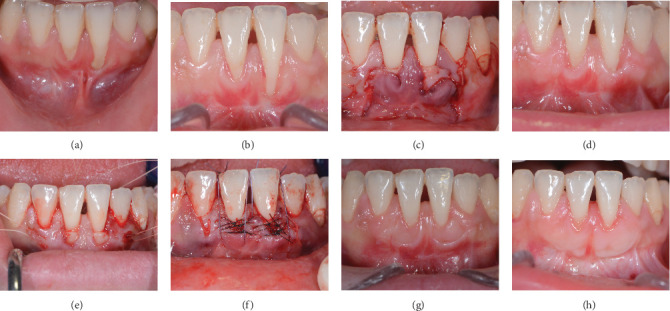
(A) Preoperative presentation. Note frenum pull and problems for correct oral hygiene. (B) Around 2 months after vestibuloplasty [24] and before root coverage procedure. Note the extensive Miller class III/RT2 recessions, thin gingiva, and lack of tension from the frenum. (C) Connective tissue graft sutured under prepared flap design utilizing the bilateral pedicle flap-tunnel technique [20, 27]. (D) Results 3 months after the first surgery and before retreatment. Note the partial root coverage and thicker tissues surrounding the remaining recessions. (E) Retreatment surgery—Connective tissue graft (presutured in situ) under prepared tunnel utilizing the modified tunnel double papilla technique (MDTP) [16]. (F) End of the second surgery with the closed tunnel and vertical mattress sutures. (G) Results 7 weeks after surgery. (H) Results 3 years after surgery. Note the almost complete root coverage with enlargement of the attached gingiva and phenotype modification.

**Table 1:** Types of first and second surgical techniques applied according to tooth number and initial Miller classification.

Tooth number	Miller class	First surgical technique	Second surgical technique
33	Cl-II	CTG + partial thickness pedicle graft [15]	CTG + partial thickness pedicle graft
31	Cl-II	MTDP [16]	MTDP
31^a^	Cl-II	MTDP	MTDP
41^a^	Cl-I	MTDP	MTDP
41	Cl-II	MTDP	CTG + partial thickness pedicle graft
31	Cl-II	MTDP	CTG + partial thickness pedicle graft
31	Cl-II	MTDP	CTG + partial thickness pedicle graft
31	Cl-II	MTDP	FGG [22]
31^b^	Cl-II	MTDP	Bilateral pedicle flap-tunnel technique [20]
41^b^	Cl-II	MTDP	Bilateral pedicle flap-tunnel technique
31	Cl-I	CTG [18]	CAF + CTG [19]
42	Cl-III	CTG + partial thickness pedicle graft	CTG
43^c^	Cl-III	MTDP	MTDP
42^c^	Cl-III	MTDP	MTDP
41	Cl-III	MTDP	CTG + partial thickness pedicle graft
31^d^	Cl-III	Bilateral pedicle flap-tunnel technique	MTDP
41^d^	Cl-III	Bilateral pedicle flap-tunnel technique	MTDP

^a^These teeth relate to the same patient.

^b^These teeth relate to the same patient.

^c^These teeth relate to the same patient.

^d^These teeth relate to the same patient.

**Table 2:** Clinical parameters assessed prior to surgical treatment and after “retreatment”.

Tooth number	Miller class	Pre- 1^st^ operation			Pre- 2^nd^ operation (“retreatment”)			After “retreatment”		
		RD (mm)	KT (mm)	AL (mm)	RD (mm)	KT (mm)	AL (mm)	RRD (mm)	KT [KTG] (mm)	AL [CAL gain] (mm)
33	II	6	0	7	4	1	5	1	3 [3]	2 [5]
31	II	7	1	8	1^e^	3	1^e^	1	3 [2]	2 [6]
31^a^	II	5	1	6	4	1	5	0	4 [3]	0 [6]
41^a^	I	2	2	3	1	1	2	0	2 [0]	0 [3]
41	II	7	1	8	7	1	8	4	2 [1]	5 [3]
31	II	4	1	5	4	1	5	0	4 [3]	1 [4]
31	II	6	1	7	3	2	4	0	4 [3]	1 [6]
31	II	4	0	5	4	1	5	1	3 [3]	1 [4]
31^b^	II	3	1	4	3	2	4	1	3 [2]	2 [2]
41^b^	II	4	1	5	4	2	5	1	3 [2]	2 [3]
31	I	2	1	3	5	2	6	0	5 [4]	1 [2]
42	III	4	0	5	4	2	5	0	5 [5]	1 [4]
43^c^	III	5	1	6	3	2	4	1	5 [4]	1 [5]
42^c^	III	7	1	8	4	2	5	0	5 [4]	1 [7]
41	III	7	1	8	6	2	7	3	2 [1]	4 [4]
31^d^	III	7	0	8	4	3	5	0	6 [6]	1 [7]
41^d^	III	3	0	4	3	3	4	0	6 [6]	1 [3]

^a^These teeth relate to the same patient.

^b^These teeth relate to the same patient.

^c^These teeth relate to the same patient.

^d^These teeth relate to the same patient.

^e^Nine months after the initial surgery, a small fenestration 4 mm apical to the free gingival margin was observed, prompting the patient to opt for a second surgical intervention.

## Data Availability

The data that support the findings of this study are available upon request from the corresponding author. The data are not publicly available due to privacy or ethical restrictions.
